# Biomechanical Evaluation of a Novel Bionic Hip Prosthesis Designed for Optimized Load Transfer and Initial Stability

**DOI:** 10.1111/os.70193

**Published:** 2025-11-06

**Authors:** Zhentao Ding, Lijia Zhang, Xingguo Wu, Xiaomeng Zhang, Chen Xiong, Yanhua Wang, Dianying Zhang

**Affiliations:** ^1^ Department of Orthopaedics and Trauma Peking University People's Hospital Beijing China; ^2^ National Centre for Trauma Medicine Peking University People's Hospital Beijing China; ^3^ Key Laboratory of Trauma and Neural Regeneration (Peking University) Ministry of Education Beijing China; ^4^ Department of Orthopaedics Peking Union Medical College Hospital Beijing China; ^5^ Department of Orthopaedics Tianjin Fifth Central Hospital Tianjin China

**Keywords:** biomechanical testing, bionic reconstruction, initial stability, stress shielding, total hip arthroplasty

## Abstract

**Objective:**

In total hip arthroplasty, the femoral component design requires a trade‐off between initial stability and stress shielding. We designed the new bionic hip arthroplasty (BHA) prosthesis with compression and tension screws to mimic compression and tension trabeculae for bionic reconstruction. This prosthesis is designed to reduce stress shielding by mimicking physiological load transfer while ensuring sufficient initial stability for successful bone integration. This study aimed to biomechanically evaluate the initial stability and migration pattern of the BHA prosthesis under dynamic and static loading conditions.

**Methods:**

The BHA prostheses were implanted into ten Sawbones fourth‐generation composite non‐osteoporotic femurs. In dynamic fatigue testing, the irreversible displacements and migration patterns in vertical and rotational directions were analyzed after 1,000,000 loading cycles. In static failure testing, the failure load of the BHA implanted model was analyzed.

**Results:**

In dynamic fatigue testing, the irreversible subsidence displacement of the BHA prosthesis was (0.3683 ± 0.1046) mm and the irreversible retroversion displacement was (0.0328 ± 0.0157)°. The irreversible displacements in both vertical and rotational directions stabilized at 100,000 loading cycles. In static failure testing, the failure load of the BHA implanted model was (4485 ± 702) N.

**Conclusions:**

The irreversible subsidence displacement of the BHA prosthesis was below the interface failure threshold of 1.5 mm, and secondary fixation was accomplished at 100,000 loading cycles. The average failure load was approximately 6.4 times body weight, much higher than the daily load range of hip joints. The BHA prosthesis suggests potential for adequate axial initial stability to facilitate bone ingrowth, which is expected to reduce revision rates in patients.

## Graphical abstract

The new bionic hip prosthesis aims to achieve bionic reconstruction of both compression and tension trabeculae by compression and tension screws. The biomechanical study confirmed that this new bionic hip prosthesis has sufficient initial stability, predicting excellent bone growth after implantation.

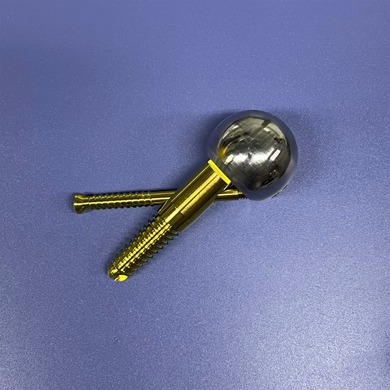

## 1 Introduction

Total hip arthroplasty (THA) has revolutionized the treatment of end‐stage hip disease, but periprosthetic osteolysis and aseptic loosening are important factors affecting long‐term survivorship [1]. Standard THA remains challenging for young and active patients because of the longer life expectancy, higher daily activity level, and higher revision probability. Short‐stem hip arthroplasty (SHA) and hip resurfacing arthroplasty (HRA) are promising attempts to optimize implant design, which effectively preserve the proximal femoral bone stock, but are still unable to completely avoid stress reduction in the femoral calcar and greater trochanter [2, 3]. Formica et al. reported a 10‐year survivorship rate of 94.8% for 194 collum femoris preserving (CFP) short stems, with a bone resorption rate of 16.1%, of which 84.4% occurred in the proximal femur [4]. Therefore, alternative surgical solutions need further exploration.

Initial stability and stress shielding are important mechanical indicators for evaluating the femoral component [5]. Previous biomechanical studies of various bone‐preserving implants have revealed that reduced stress shielding implies less mechanical stimulation to the cortical bone [6]. It also leads to elevated stress distribution at the implant‐bone interface, which affects the initial stability [7]. Consequently, the ideal femoral component needs to balance fixation failure due to insufficient initial stability and bone loss due to stress shielding.

**FIGURE 1:**
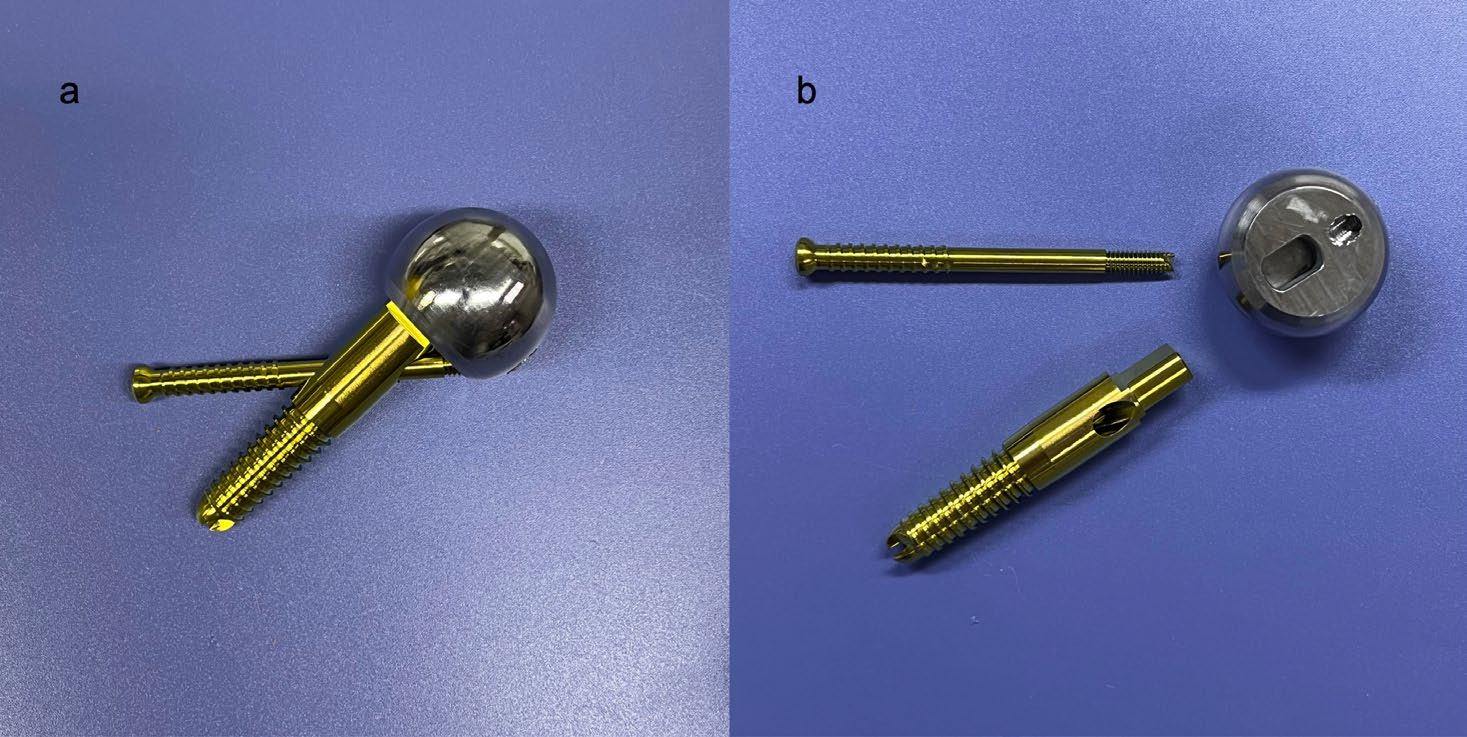
Anterior profile of the BHA prosthesis. (a) Assembled BHA prosthesis. (b) The compression screw is tapered to the prosthetic head, and the tension screw is threaded and locked to the prosthetic head.

The main purpose of femoral component design is to restore the biomechanical architecture of hip joints, which is also known as the bionic reconstruction concept [8]. Based on this, we designed a new hip prosthesis. The compression screw is designed to mimic compression trabeculae and the tension screw is designed to mimic tension trabeculae with connection through a prosthetic head. This prosthesis aims to achieve bionic reconstruction of both compression and tension trabeculae, hence the name bionic hip arthroplasty (BHA) prosthesis (Figure 1a). Compared to standard THA and SHA, the novel BHA prosthesis features shorter femoral component length and more proximal fixation, allowing for increased proximal loads and decreased distal loads after implantation. Under loading conditions, the compression screw tends to be compressed and the tension screw tends to be tensioned, resulting in a more physiological load transfer pattern in the proximal femur, thereby reducing stress shielding and providing adequate initial stability. However, this preliminary study lacks direct comparisons with existing short‐stem or standard‐stem prostheses, and the claim of reduced stress shielding is derived from theoretical design principles requiring further experimental and clinical validation.

The objective of this study was to evaluate the initial stability of BHA prosthesis by biomechanical testing and to investigate the migration pattern after implantation. Excellent initial stability is a prerequisite for bone ingrowth and is essential for the long‐term stability of uncemented prostheses [9]. Initial stability can be quantified by the irreversible displacement in dynamic fatigue testing [10, 11]. Irreversible displacement greater than 1.5 mm within 2 years of implantation suggests a higher rate of prosthesis revision [12]. In addition, the failure load in static failure testing can also predict the stability performance of prostheses under extreme loading conditions.

## 2 Methods

### 2.1 Implant Design

The BHA prosthesis is designed with a combination of compression screw, tension screw, and prosthetic head. We assume that when the screw angle is aligned with the trabecular orientation, the screw is able to mimic the biomechanical properties of the trabecular bone. The axis of the compression screw is parallel to the axis of the femoral neck and therefore at 135° to the axis of the femoral shaft. Our previous study found that the proximal femur demonstrated the most physiological stress distribution when the tension screw was at 95° to the axis of the femoral shaft [13]. For this reason, the BHA prosthesis adopts the same tension screw angle setting. The compression screw is tapered to the prosthetic head with an eccentric distance of 4 mm for larger joint mobility. The head end of the tension screw is located at the innominate tubercle, a bony prominence lateral to the greater trochanter [14]. The tension screw passes through the central hole of the compression screw, and the tail end is threaded and locked to the prosthetic head (Figure 1b).

### 2.2 Model Preparation

Ten left medium Sawbones fourth‐generation composite femurs (Model 3403, Pacific Research Laboratories, Vashon, WA, USA) were prepared, all of which were non‐osteoporotic models. The diameters of compression screws include 13 mm, 13.5 mm, 14 mm, 14.5 mm, and 15 mm. The diameters of tension screws include 5 mm, 5.5 mm, 6 mm, and 6.5 mm. The appropriate size of the BHA prosthesis was selected based on the synthetic femur. The diameter of the compression screw was chosen as 14 mm, and the diameter of the tension screw was chosen as 5.5 mm. The material of the prosthesis is titanium alloy.

**FIGURE 2:**
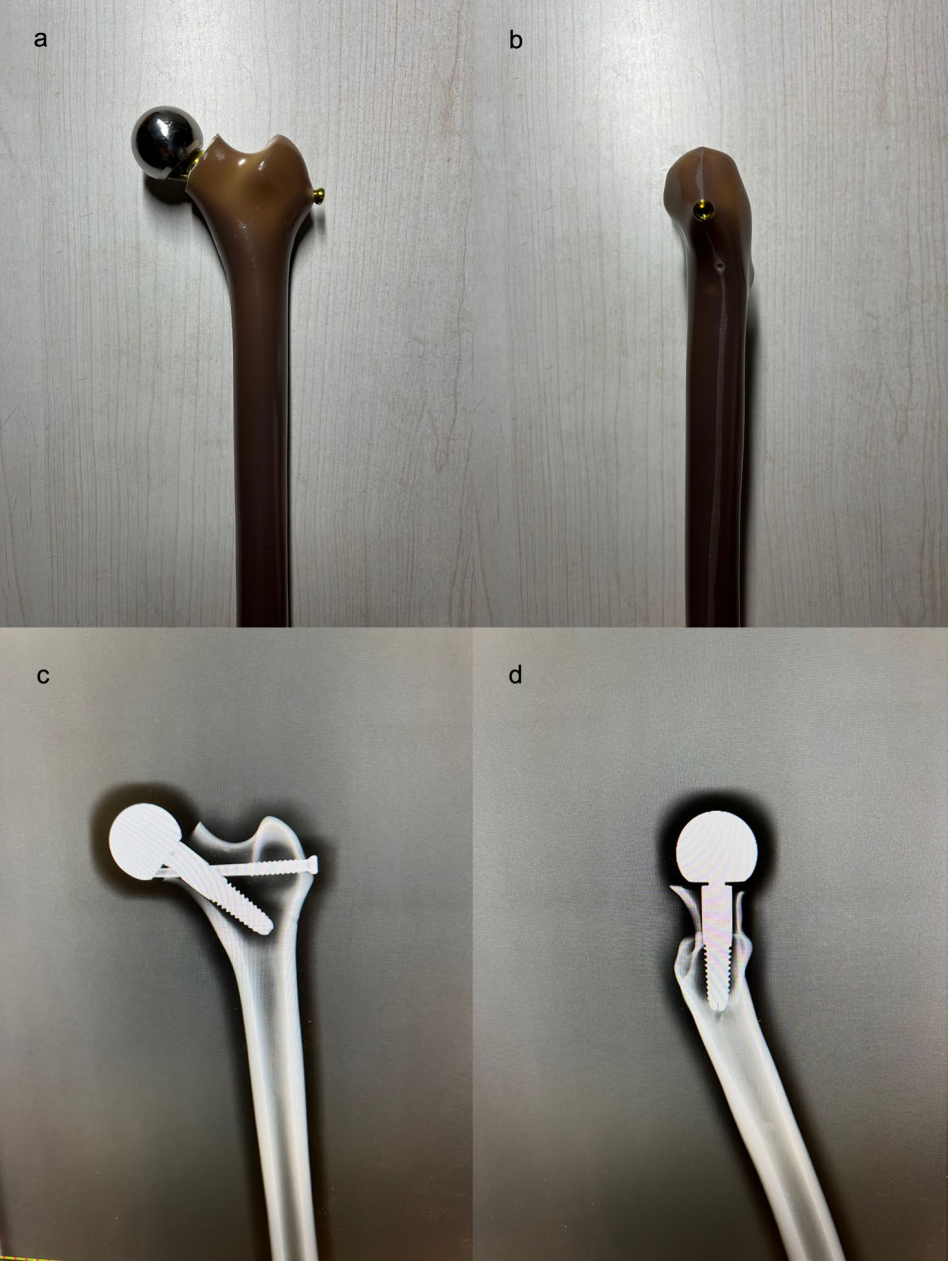
BHA implanted model preparation. (a) Frontal and (b) lateral profiles of BHA implanted model. (c) Frontal and (d) lateral radiographs of BHA implanted model.

The same senior orthopedic surgeon performed all the implantations. The synthetic femur was osteotomized according to the collum osteotomy level [15]. A guide pin was inserted 4 mm below the center of the osteotomy surface along the axis of the femoral neck, which was 135° to the axis of the femoral shaft. Drilling was performed in the direction of the guide pin to a depth that reached the medial aspect of the femoral lateral cortex without penetrating. A compression screw was then inserted and connected to the prosthetic head. Next, a guiding device for the tension screw was threaded onto the prosthetic head. Another guide pin was inserted along the guiding device and through the prosthetic head, at 95° to the axis of the femoral shaft. Drilling was performed in the direction of the guide pin to break through the cortical bone of the innominate tubercle, followed by reverse insertion of a tension screw. The tension screw was threaded into the prosthetic head to ensure locking. The profiles of the BHA implanted model are shown in Figure 2a,b. X‐ray fluoroscopy (GE Healthcare, Madison, WI, USA) was applied to determine the position of the prostheses (Figure 2c,d).

The BHA implanted model was tilted 10° laterally in the coronal plane and 9° posteriorly in the sagittal plane to simulate a one‐legged stance (Georg [16]). The distal femur was osteotomized for 10 cm. The distal end of the implanted model was fixed in a steel cup with methyl methacrylate and embedded to a depth of 6 cm.

### 2.3 Dynamic Fatigue Testing

**FIGURE 3:**
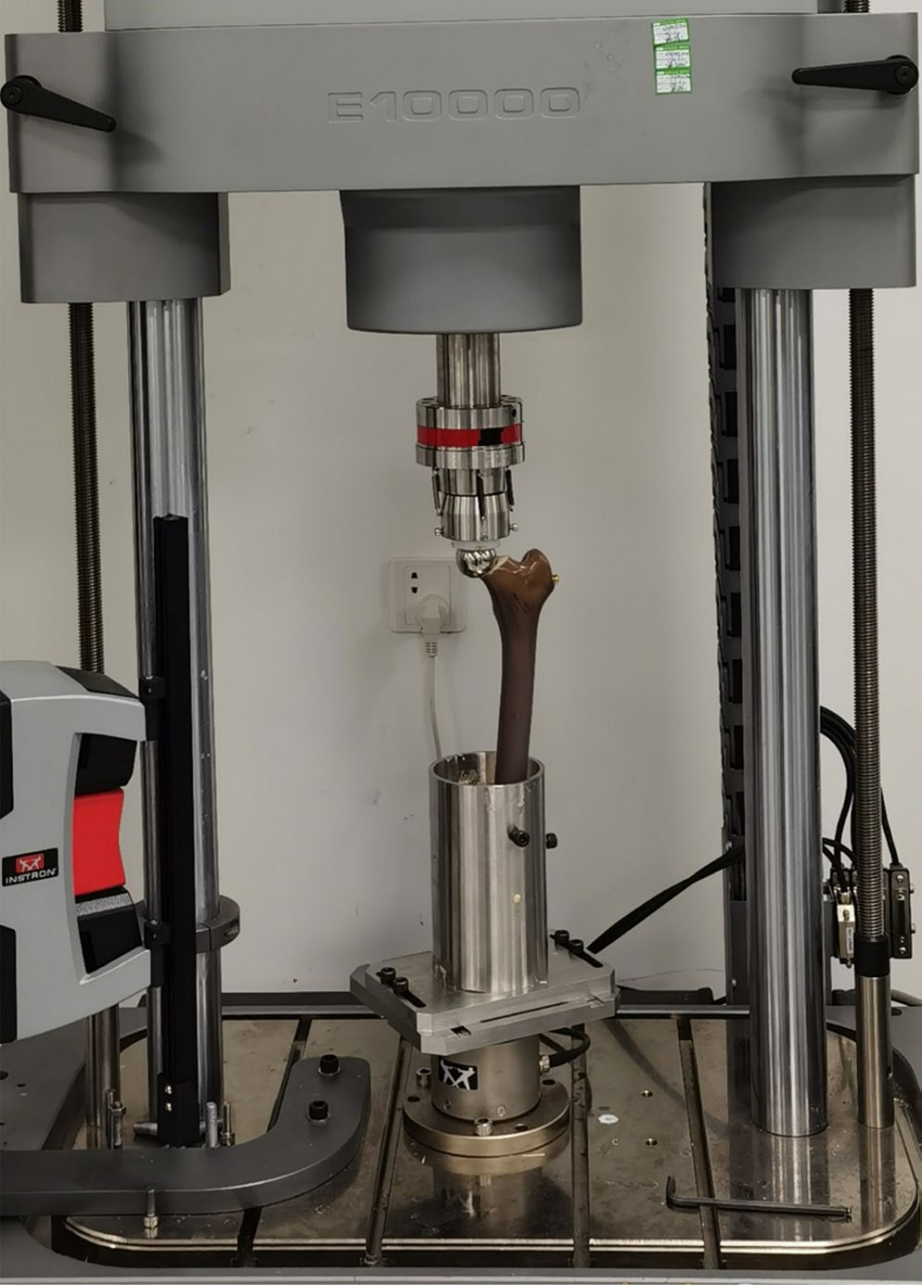
Biomechanical testing setup.

Five BHA implanted models were prepared for dynamic fatigue testing. The implanted models were immobilized in a servo‐hydraulic biomechanical testing machine (ElectroPuls E10000, Instron Systems, Norwood, MA, USA) (Figure 3) with a range of 10,000 N. A vertical load was applied to the top of the prosthetic head at a frequency of 2 Hz. The load range was 160–1600 N, equivalent to 2.5 times body weight, which simulated the hip joint loading during normal walking gait (Graichen [17]). A total of 1,000,000 cycles were dynamically loaded, equivalent to simulating the walking activity for 1 year [18]. Vertical displacement and rotational angle were recorded every 1000 cycles with a precision of 1 μm and 1 mdeg, respectively, as measured by sensors on the loading device. While this method provides valuable data on irreversible displacement under controlled conditions, it should be noted that Roentgen Stereophotogrammetric Analysis (RSA) is considered the gold standard for quantifying three‐dimensional micromotion of orthopedic implants in both clinical and preclinical settings [19]. Comparison of relative positions before and after dynamic loading allowed analysis of the irreversible displacements in vertical and rotational directions. Plotting displacement‐cycle curves allowed analysis of the migration pattern. The irreversible displacement of the prosthesis would stabilize after secondary fixation.

### 2.4 Static Failure Testing

Five BHA implanted models were prepared for static failure testing. The implanted models were immobilized in the same biomechanical testing machine. A vertical load was applied to the top of the prosthetic head at a loading speed of 5 mm/min until fracture occurred in the synthetic femoral model or BHA prosthesis. Plot the load–displacement curves and record the failure load and maximum displacement. The linear segment in the load–displacement curve was selected to calculate the overall stiffness of the implanted model.

### 2.5 Statistical Analysis

Statistical analysis and graphing were performed using GraphPad Prism 9 (GraphPad Software, San Diego, USA). The Shapiro–Wilk test was applied for normality testing. Continuous variables that follow the normal distribution used mean and standard deviation to describe the data distribution.

## 3 Results

### 3.1 Irreversible Displacement

In dynamic fatigue testing, no fracture occurred in all BHA implanted models during 1,000,000 loading cycles. The irreversible subsidence displacement of the BHA prosthesis in the vertical direction was (0.3683 ± 0.1046) mm. The irreversible retroversion displacement in the rotational direction was (0.0328 ± 0.0157)°. The irreversible displacements of each BHA prosthesis in vertical and rotational directions are shown in Table 1.

**TABLE 1:** Irreversible displacements of BHA prosthesis in vertical and rotational directions.

Number	Vertical irreversible displacement (mm)	Rotational irreversible displacement (°)
#1	0.5382	0.0353
#2	0.3898	0.0515
#3	0.2697	0.0088
#4	0.3365	0.0289
#5	0.3073	0.0394

### 3.2 Migration Pattern

**FIGURE 4:**
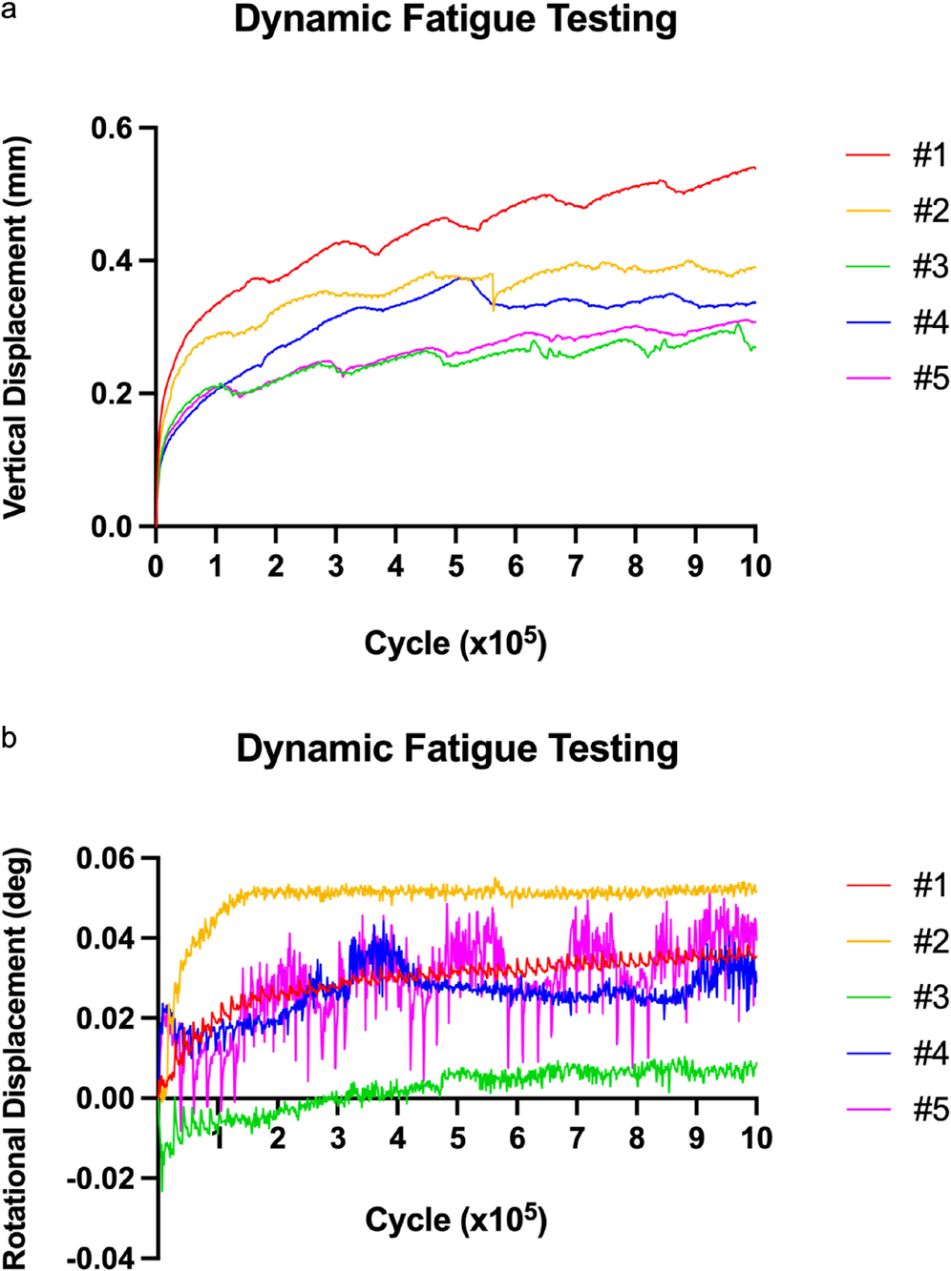
Dynamic fatigue testing. (a) Vertical displacement‐cycle curves and (b) rotational displacement‐cycle curves show the subsidence and retroversion displacement of five BHA implanted models over 1,000,000 cycles.

In dynamic fatigue testing, vertical displacement‐cycle curves of each BHA prosthesis are shown in Figure 4a, and rotational displacement‐cycle curves are shown in Figure 4b. The irreversible displacements in both vertical and rotational directions stabilized at 100,000 loading cycles.

The irreversible subsidence displacement of BHA prosthesis was (0.2490 ± 0.0583) mm in 100,000 cycles before the transition point. The irreversible subsidence displacement was (0.1193 ± 0.0545) mm in 900,000 cycles after the transition point. The irreversible retroversion displacement of BHA prosthesis was (0.0170 ± 0.0194)° in 100,000 cycles before the transition point. The irreversible retroversion displacement was (0.0158 ± 0.0070)° in 900,000 cycles after the transition point.

### 3.3 Failure Load

**FIGURE 5:**
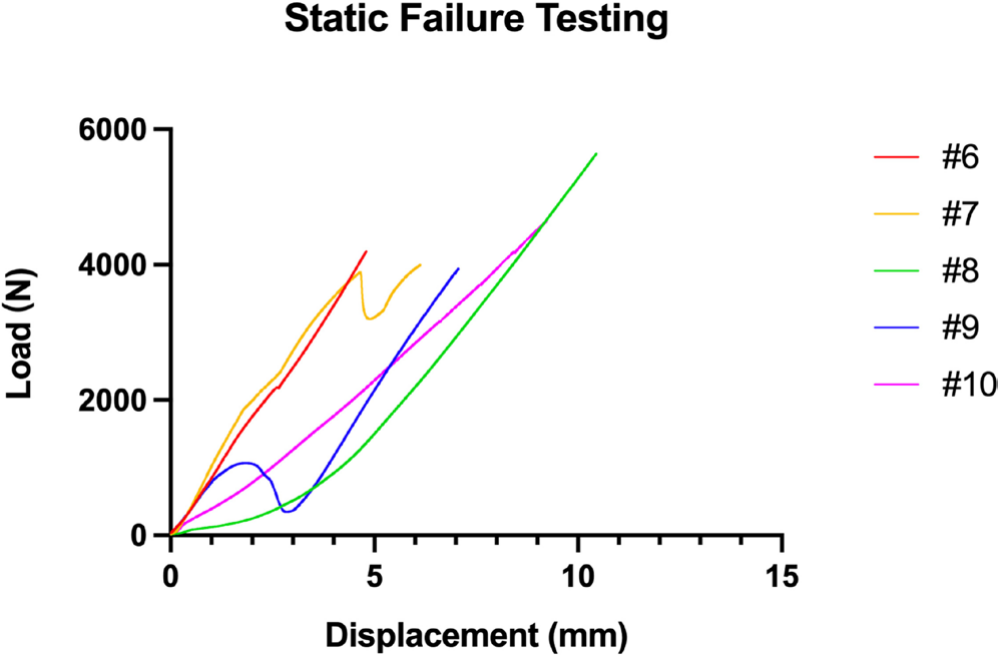
Static failure testing. Load–displacement curves show the failure load, maximum displacement, and stiffness of five BHA implanted models.

In static failure testing, the failure load of the BHA implanted model was (4485 ± 702) N, the maximum displacement was (7.54 ± 2.29) mm, and the stiffness was (779.3 ± 157.4) N/mm. The data for each implanted model is shown in Table 2. The load–displacement curves are shown in Figure 5.

**TABLE 2:** Failure load, maximum displacement and stiffness of BHA implanted models.

Number	Failure load (N)	Maximum displacement (mm)	Stiffness (N/mm)
#6	4199.98	4.81	853.24
#7	3997.63	6.13	854.75
#8	5640.26	10.45	764.48
#9	3941.73	7.06	910.52
#10	4643.75	9.23	513.69

### 3.4 Failure Pattern

**FIGURE 6:**
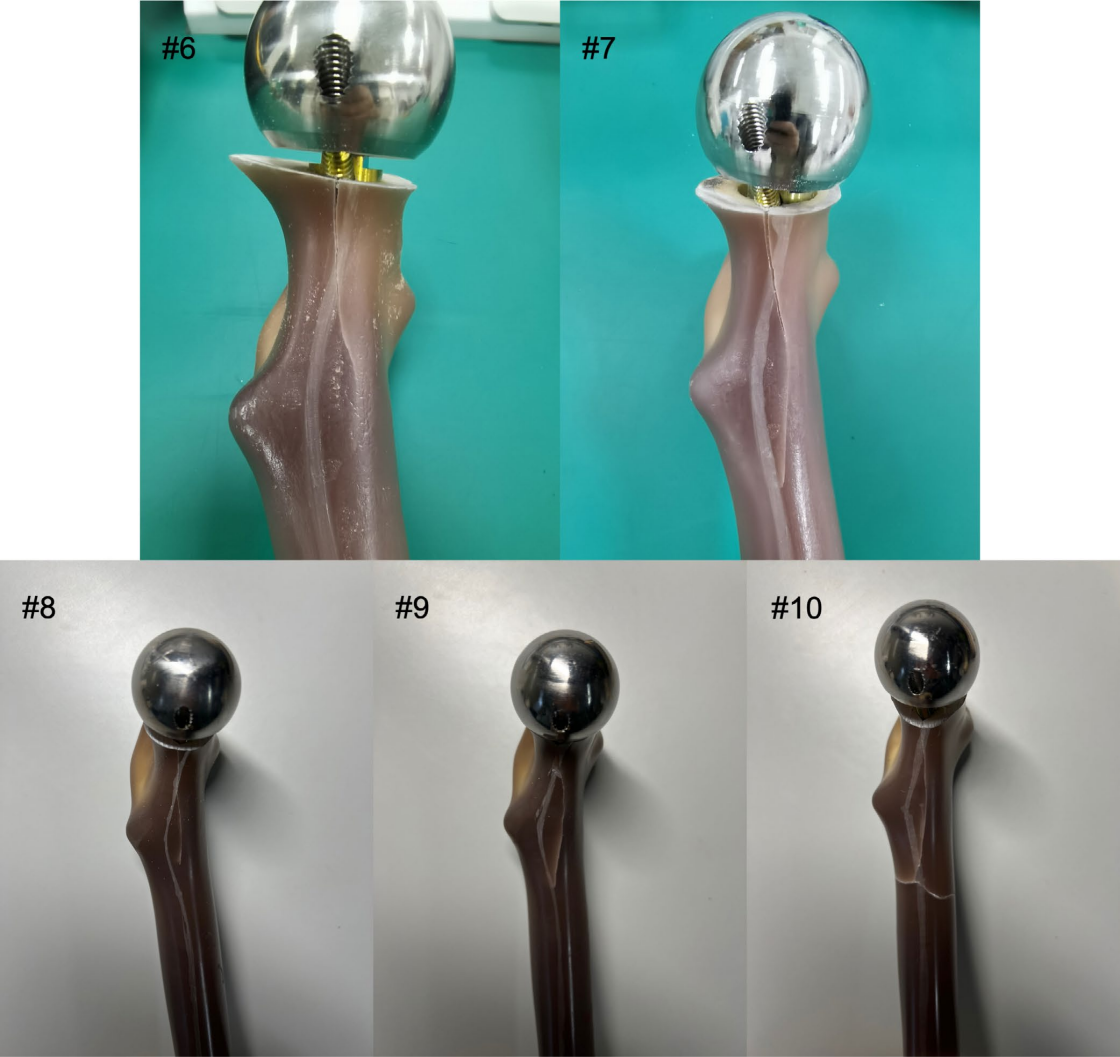
Failure pattern of BHA implanted models in static failure testing. The fracture sites of all five models were the medial cortex of the femoral neck beneath compression screws.

In static failure testing, the failure pattern of each BHA implanted model is shown in Figure 6. The fracture sites of all five implanted models were the medial cortex of the femoral neck beneath compression screws. The fracture lines extended distally along the medial femoral neck. In the #10 implanted model, the fracture line extended from the medial femoral neck to the subtrochanteric region and formed a transverse fracture line.

## 4 Discussion

In this study, the axial initial stability of the BHA prosthesis was evaluated by dynamic fatigue testing and static failure testing. The irreversible subsidence displacement of the BHA prosthesis was below the interface failure threshold, and secondary fixation was accomplished at 100,000 loading cycles. The failure load of the BHA implanted model was much higher than the daily load range of hip joints.

### 4.1 Irreversible Displacement

In a clinical study, Karrholm et al. found that the revision rate could be as high as 50% when irreversible displacement was greater than 1.5 mm within 2 years of implantation, thus determining the interface failure threshold [12]. Irreversible subsidence displacement varied considerably across short and standard stems in multiple follow‐up reports, but all were below this threshold [20, 21]. Bieger et al. implanted Fitmore short stem, Mayo short stem, and CLS standard stem into cadaveric femur specimens and dynamically loaded them for 100,000 cycles with a load range of 100–1600 N [22]. The average subsidence displacement was found to be 0.105 mm for Fitmore short stem, 0.248 mm for Mayo short stem, and 0.526 mm for CLS standard stem. Compared to existing short and standard stems, the BHA prosthesis achieved subsidence displacement (0.3683 mm) within the range reported for clinically successful implants (0.105–0.526 mm), while simultaneously reducing coronal dimension to minimize stress shielding. This underscores its potential to balance stability and physiological load transfer. However, this study did not include direct biomechanical or clinical comparisons with existing short‐stem or standard‐stem prostheses, which limits the ability to assert its superiority. Regardless, the irreversible subsidence displacement of the BHA prosthesis was below the interface failure threshold of 1.5 mm, suggesting that the BHA prosthesis has potential for adequate axial initial stability to facilitate bone ingrowth. Future comparative studies are warranted to validate these theoretical advantages against established designs.

Engh et al. discovered in an imaging study that the femoral component with smaller coronal and sagittal dimensions has a higher incidence of prosthetic subsidence, and therefore recommended that the design should be adjusted in the proximal region to improve initial stability [23]. Biomechanical investigations suggested that stem length shortening within a certain range does not affect the initial stability [24, 25]. Moreover, the proximal fixation improves initial stability and compensates for the adverse effects of shorter stem lengths [22]. The coronal dimension of the BHA prosthesis is further shortened compared to short and standard stems, aiming to reduce the stress shielding after implantation. The biomechanical testing showed that the BHA prosthesis still maintained good axial initial stability, which may be due to the bionic reconstruction of both compression and tension trabeculae by compression and tension screws, so that the stress distribution in the proximal femur is closer to physiological conditions. As a result, the BHA prosthesis is able to achieve a trade‐off between lower stress shielding and adequate initial stability. The bionic reconstruction concept aims to mimic the natural load transfer pathways of the proximal femur. The compression and tension screws are intended to share load in a manner analogous to the physiological trabeculae. However, it should be noted that this study provides only indirect mechanical stability evidence for the bionic concept and the proposed reduction in stress shielding. It does not offer direct proof of its efficacy in restoring physiological loading or reducing stress shielding. Therefore, these claims remain theoretical and require further experimental confirmation through direct strain measurements or finite element analysis to quantify load sharing and proximal femoral strain distribution.

Rotational initial stability is also an important factor influencing the process of bone growth. Since prosthetic rotation usually occurs in the proximal region, the proximal fixed prosthesis may have superior rotational stability [26]. The irreversible retroversion displacement of the BHA prosthesis (0.0328°) was much lower than the results of short‐stem (0.2°) and standard‐stem (0.3°) in other studies [22]. However, no reports have specified the interface failure threshold for rotational displacement.

### 4.2 Migration Pattern

There is typically a secondary fixation process occurring with uncemented prostheses. Primary fixation is accomplished at the time of implantation. Walking activity compacts the cancellous bone around the prosthesis and achieves full contact, at which point secondary fixation is accomplished [27]. After the transition point of secondary fixation, the irreversible displacement stabilizes. Bieger et al. demonstrated by biomechanical testing of cadaveric femur specimens that the irreversible displacement of short and standard stems stabilized at 3000–8000 loading cycles [28]. Westphal et al. similarly observed the transition point to be 4000 loading cycles [29]. The irreversible displacements of BHA prosthesis in both vertical and rotational directions stabilized at 100,000 loading cycles, equivalent to 6 weeks of walking activity [18]. It is important to note that this stabilization represents the mechanical integration phase of the implant‐bone interface in a synthetic model, whereas clinical secondary fixation in vivo involves biological processes such as bone ingrowth and remodeling, which typically progress over 3 to 6 months postoperatively [20]. Therefore, the temporal relationship between synthetic model outcomes and clinical timelines should be interpreted cautiously due to inherent differences in biological adaptation.

### 4.3 Failure Load

Sawbones composite model has a high degree of consistency and reproducibility [30]. In biomechanical investigations, the Sawbones non‐osteoporotic femur model had an average failure load of 2895.1 N and an average axial stiffness of 555.1 N/mm, whereas the cadaveric femur specimen under the same conditions had an average failure load of 3316.2 N and an average axial stiffness of 419.2 N/mm [31, 32]. In this study, the failure load and axial stiffness of the BHA implanted model were obviously higher than those of the composite femur model without implantation. This indicates that the BHA prosthesis provides sufficient axial initial stability to resist axial compressive load, and no fracture of the femur or prosthesis may occur under daily conditions.

Taylor et al. measured hip forces of 0.9–1.3 times body weight in two‐legged stance and 1.8–2.3 times body weight in one‐legged stance [33]. Assuming an average body weight of 70 kg for adult males, the average failure load of the BHA implanted model was 6.4 times body weight, much higher than the daily load range of hip joints. However, the BHA implanted model may still fracture under extreme loading conditions or when the hip joint is subjected to shear stress.

### 4.4 Limitations

This study provides the first biomechanical evaluation of a novel bionic hip prosthesis design under standardized testing conditions. The use of controlled synthetic models and routine cyclic loading protocols ensures the reproducibility of the stability data, forming a critical foundation for future comparative and clinical studies.

However, translating in vitro results into in vivo results can be challenging. The use of Sawbones composite femurs, while ensuring experimental reproducibility, inherently lacks biological variability such as differences in bone density, osteoporotic conditions, and adaptive bone remodeling processes. While composite models provide standardized testing conditions, future studies utilizing cadaveric specimens or patient‐specific finite element models will further elucidate the prosthesis's performance in biological tissues with varying bone densities and geometries. Besides, the displacement sensors were located on the loading device and could not analyze the reversible micromotion of the implant‐bone interface to assess initial stability from another perspective. It should be noted that the dynamic fatigue testing focused solely on vertical loading conditions, ignoring torsional and shear forces critical to hip joint biomechanics, which is a limitation of the current experimental setup. All BHA prostheses in this study were positioned ideally within the femur models, and the effects of varus and valgus on initial stability were not explored. In addition, the BHA prosthesis requires high surgical precision. Achieving consistent implant positioning may be challenging for different surgeons; therefore, specialized training and guiding instruments will help to improve surgical reproducibility.

### 4.5 Prospects of Clinical Application

Despite these limitations, the BHA prosthesis holds significant clinical potential for young and active patients requiring hip arthroplasty, as its bionic design aims to reduce stress shielding while maintaining initial stability. However, clinical translation may face challenges such as surgical learning curves, long‐term bone remodeling responses, and patient‐specific anatomical variations. Future work should focus on conducting cadaveric validations, developing surgical navigation tools, and initiating controlled clinical trials to evaluate its in vivo performance and longevity.

## 5 Conclusions

The design of the BHA prosthesis is based on the bionic reconstruction concept, which attempts to trade off initial stability and stress shielding. This in vitro study confirmed by dynamic fatigue testing and static failure testing that the BHA prosthesis has sufficient axial initial stability to avoid fixation failure at the implant‐bone interface after implantation. However, the practical application of hip prostheses requires rigorous preclinical evaluation, and the mechanical properties still need further validation and improvement. Future investigations will include multiaxial fatigue testing beyond 1 million cycles to evaluate long‐term mechanical integrity under physiologically relevant conditions. Scanning electron microscopy (SEM) will be utilized to characterize fatigue crack initiation and propagation, providing mechanistic insights into failure modes. These studies will complement ongoing clinical trials monitoring in vivo performance and bone remodeling patterns.

## Author Contributions

**Zhentao Ding:** data curation, formal analysis, methodology, visualization, writing – original draft, writing – review and editing. **Lijia Zhang:** conceptualization, resources, writing – review and editing. **Xingguo Wu:** methodology, software, writing – review and editing. **Xiaomeng Zhang:** investigation, methodology. **Chen Xiong:** investigation, methodology. **Yanhua Wang:** data curation, funding acquisition, supervision, writing – review and editing. **Dianying Zhang:** conceptualization, funding acquisition, supervision, writing – review and editing.

## Ethics Statement

This study was approved by the institutional review board of Peking University People's Hospital Ethics Review Committee (2022PHB237‐001) and was performed in accordance with the Declaration of Helsinki.

## Consent

The informed consent form was exempted in this biomechanical study.

## Conflicts of Interest

The authors declare no conflicts of interest.

## References

[1] Z. Shao and S. Bi , “Patient Satisfaction After Total Hip Arthroplasty: Influencing Factors,” Frontiers in Surgery 9 (2023): 1043508. 36793514 10.3389/fsurg.2022.1043508 PMC9922864

[2] J. Girard , A. Lons , N. Ramdane , and S. Putman , “Hip Resurfacing Before 50 Years of Age: A Prospective Study of 979 Hips With a Mean Follow‐Up of 5.1 Years,” Orthopaedics & Traumatology, Surgery & Research 104, no. 3 (2018): 295–299. 10.1016/j.otsr.2017.10.018 29277514

[3] S. Lidder , D. Epstein , and G. Scott , “A Systematic Review of Short Metaphyseal Loading Cementless Stems in Hip Arthroplasty,” Bone & Joint Journal 101, no. 5 (2019): 502–511. 31037973 10.1302/0301-620X.101B5.BJJ-2018-1199.R1

[4] M. Formica , L. Cavagnaro , M. Basso , A. Zanirato , A. Palermo , and L. Felli , “What Is the Fate of the Neck After a Collum Femoris Preserving Prosthesis? A Nineteen Years Single Center Experience,” International Orthopaedics 41, no. 7 (2017): 1329–1335. 27889839 10.1007/s00264-016-3350-9

[5] D. Vogel , M. Wehmeyer , M. Kebbach , H. Heyer , and R. Bader , “Stress and Strain Distribution in Femoral Heads for Hip Resurfacing Arthroplasty With Different Materials: A Finite Element Analysis,” Journal of the Mechanical Behavior of Biomedical Materials 113 (2021): 104115. 33189013 10.1016/j.jmbbm.2020.104115

[6] D. Savio and A. Bagno , “When the Total Hip Replacement Fails: A Review on the Stress‐Shielding Effect,” PRO 10, no. 3 (2022): 612.

[7] M. L. Raffa , V. H. Nguyen , P. Hernigou , C. H. Flouzat‐Lachaniette , and G. Haiat , “Stress Shielding at the Bone‐Implant Interface: Influence of Surface Roughness and of the Bone‐Implant Contact Ratio,” Journal of Orthopaedic Research 39, no. 6 (2021): 1174–1183. 32852064 10.1002/jor.24840

[8] N. Santori , M. Lucidi , and F. Santori , “Proximal Load Transfer With a Stemless Uncemented Femoral Implant,” Journal of Orthopaedics and Traumatology 7 (2006): 154–160.

[9] J. van Loon , A. Vervest , H. van der Vis , et al., “Ceramic‐On‐Ceramic Articulation in Press‐Fit Total Hip Arthroplasty as a Potential Reason for Early Failure, What About the Survivors: A Ten Year Follow‐Up,” International Orthopaedics 45 (2021): 1447–1454. 33459828 10.1007/s00264-020-04895-1 PMC8178149

[10] D. Bühler , U. Berlemann , K. Lippuner , P. Jaeger , and L. Nolte , “Three‐Dimensional Primary Stability of Cementless Femoral Stems,” Clinical Biomechanics 12, no. 2 (1997): 75–86. 11415676 10.1016/s0268-0033(96)00059-9

[11] C. A. Engh , D. O'Connor , M. Jasty , T. F. McGovern , J. D. Bobyn , and W. H. Harris , “Quantification of Implant Micromotion, Strain Shielding, and Bone Resorption With Porous‐Coated Anatomic Medullary Locking Femoral Prostheses,” Clinical Orthopaedics and Related Research 285 (1992): 13–29. 1446429

[12] J. Karrholm , B. Borssen , G. Lowenhielm , and F. Snorrason , “Does Early Micromotion of Femoral Stem Prostheses Matter? 4‐7‐Year Stereoradiographic Follow‐Up of 84 Cemented Prostheses,” Journal of Bone & Joint Surgery. British Volume 76, no. 6 (1994): 912–917. 7983118

[13] Z. Ding , J. Wang , Y. Wang , X. Zhang , Y. Huan , and D. Zhang , “Bionic Reconstruction of Tension Trabeculae in Short‐Stem Hip Arthroplasty: A Finite Element Analysis,” BMC Musculoskeletal Disorders 24, no. 1 (2023): 89. 36732725 10.1186/s12891-023-06205-3 PMC9893650

[14] M. H. Ward , R. L. Williams , J. Bekvalac , S. Bajada , M. Maheson , and P. J. Adds , “The Innominate Tubercle of the Femur Is a Consistent Surgical Landmark and Shows no Variation Between Sex and Side: An Osteological Study,” Clinical Anatomy 34, no. 5 (2021): 742–747. 33347646 10.1002/ca.23714

[15] F. Falez , F. Casella , and M. Papalia , “Current Concepts, Classification, and Results in Short Stem Hip Arthroplasty,” Orthopedics 38, no. 3 (2015): S6–S13. 25826635 10.3928/01477447-20150215-50

[16] G. Bergmann , A. Bender , J. Dymke , G. Duda , and P. Damm , “Standardized Loads Acting in Hip Implants,” PLoS One 11, no. 5 (2016): e0155612. 27195789 10.1371/journal.pone.0155612 PMC4873223

[17] G. Bergmann , F. Graichen , and A. Rohlmann , “Hip Joint Loading During Walking and Running, Measured in Two Patients,” Journal of Biomechanics 26, no. 8 (1993): 969–990. 8349721 10.1016/0021-9290(93)90058-m

[18] T. P. Schmalzried , E. F. Shepherd , F. J. Dorey , et al., “Wear Is a Function of Use, Not Time,” Clinical Orthopaedics and Related Research 381 (2000): 36–46. 10.1097/00003086-200012000-00005 11127668

[19] G. Selvik , “Roentgen Stereophotogrammetry: A Method for the Study of the Kinematics of the Skeletal System,” Acta Orthopaedica Scandinavica 60, no. sup232 (1989): 1–51. 2686344

[20] Y. P. Acklin , R. Jenni , H. Bereiter , C. Thalmann , and K. Stoffel , “Prospective Clinical and Radiostereometric Analysis of the Fitmore Short‐Stem Total Hip Arthroplasty,” Archives of Orthopaedic and Trauma Surgery 136 (2016): 277–284. 26739137 10.1007/s00402-015-2401-9

[21] O. Wolf , P. Mattsson , J. Milbrink , S. Larsson , and H. Mallmin , “Periprosthetic Bone Mineral Density and Fixation of the Uncemented CLS Stem Related to Different Weight Bearing Regimes: A Randomized Study Using DXA and RSA in 38 Patients Followed for 5 Years,” Acta Orthopaedica 81, no. 3 (2010): 286–291. 20446828 10.3109/17453674.2010.487238 PMC2876828

[22] R. Bieger , A. Ignatius , R. Decking , L. Claes , H. Reichel , and L. Dürselen , “Primary Stability and Strain Distribution of Cementless Hip Stems as a Function of Implant Design,” Clinical Biomechanics 27, no. 2 (2012): 158–164. 21889243 10.1016/j.clinbiomech.2011.08.004

[23] C. A. Engh and P. Massin , “Cementless Total Hip Arthroplasty Using the Anatomic Medullary Locking Stem: Results Using a Survivorship Analysis,” Clinical Orthopaedics and Related Research 249 (1989): 141–158. 2582665

[24] S. R. Small , S. E. Hensley , P. L. Cook , et al., “Characterization of Femoral Component Initial Stability and Cortical Strain in a Reduced Stem‐Length Design,” Journal of Arthroplasty 32, no. 2 (2017): 601–609. 27597431 10.1016/j.arth.2016.07.033

[25] G. Yamako , E. Chosa , K. Totoribe , S. Watanabe , and T. Sakamoto , “Trade‐Off Between Stress Shielding and Initial Stability on an Anatomical Cementless Stem Shortening: In‐Vitro Biomechanical Study,” Medical Engineering & Physics 37, no. 8 (2015): 820–825. 26117334 10.1016/j.medengphy.2015.05.017

[26] D. Nunn , M. Freeman , K. Tanner , and W. Bonfield , “Torsional Stability of the Femoral Component of Hip Arthroplasty. Response to an Anteriorly Applied Load,” Journal of Bone & Joint Surgery British Volume 71, no. 3 (1989): 452–455. 2722940 10.1302/0301-620X.71B3.2722940

[27] P. Diehl , M. Haenle , P. Bergschmidt , et al., “Cementless Total Hip Arthroplasty: A Review,” Biomedizinische Technik. Biomedical Engineering 55, no. 5 (2010): 251–264. 20958235 10.1515/BMT.2010.037

[28] R. Bieger , A. Ignatius , H. Reichel , and L. Dürselen , “Biomechanics of a Short Stem: In Vitro Primary Stability and Stress Shielding of a Conservative Cementless Hip Stem,” Journal of Orthopaedic Research 31, no. 8 (2013): 1180–1186. 23553802 10.1002/jor.22349

[29] F. Westphal , N. Bishop , M. Honl , E. Hille , K. Püschel , and M. Morlock , “Migration and Cyclic Motion of a New Short‐Stemmed Hip Prosthesis – A Biomechanical In Vitro Study,” Clinical Biomechanics 21, no. 8 (2006): 834–840. 16806616 10.1016/j.clinbiomech.2006.04.004

[30] S. A. Naghavi , C. Lin , C. Sun , et al., “Stress Shielding and Bone Resorption of Press‐Fit Polyether–Ether–Ketone (PEEK) Hip Prosthesis: A Sawbone Model Study,” Polymers 14, no. 21 (2022): 4600. 36365594 10.3390/polym14214600 PMC9657056

[31] C. Gluek , R. Zdero , and C. E. Quenneville , “Evaluating the Mechanical Response of Novel Synthetic Femurs for Representing Osteoporotic Bone,” Journal of Biomechanics 111 (2020): 110018. 32891014 10.1016/j.jbiomech.2020.110018

[32] A. D. Heiner , “Structural Properties of Fourth‐Generation Composite Femurs and Tibias,” Journal of Biomechanics 41, no. 15 (2008): 3282–3284. 18829031 10.1016/j.jbiomech.2008.08.013

[33] S. Taylor and P. Walker , “Forces and Moments Telemetered From Two Distal Femoral Replacements During Various Activities,” Journal of Biomechanics 34, no. 7 (2001): 839–848. 11410168 10.1016/s0021-9290(01)00042-2

